# New Modes of Applying the Actual Cautery

**Published:** 1858-04

**Authors:** 


*New Modes of Applying the Actual Cautery.*—In a late number of the *Union Medicale* we find some suggestions respecting the employment of the actual cautery, which appear valuable for a class of cases in which it is not desirable to have recourse to the severe application of the old method by means of the red hot iron. M. Bonnafond has invented a new "caustic crayon," composed of 75 grains of gum tragacanth, 4 drachms of powdered wood charcoal, and 30 grains of nitrate of potass The gum is to be dissolved in a sufficient quantity of water to make a thick solution, the process being facilitated by the addition of a little sugar; then the nitre and charcoal, mixed together, are to be added gradually, to form a mass sufficiently consistent to be made into cylinders of different sizes. He afterward found that the nitre might be advantageously omitted.—The end of the cylinder being ignited, is to be lightly applied to different points of the affected part.

In a subsequent number of the same journal, Dr. Chevillion describes a still more simple method of applying the actual cautery. He employs a small roll of linen, one end of which is to be ignited in the flame of a candle. On blowing gently upon the lighted end a few moments, a little red-hot cone is formed, with which the skin is to be touched. In this way fifty or a hundred applications can be made in a few minutes. This method is especially useful in dorsal and lumbar neuralgia, sciatica, old glandular swellings, obstinate facial paralysis, dropsical effusions into the joints, &c·—*Boston Med. &. Surg, Journal.*

